# Organ transcriptomes of the lucinid clam *Loripes orbiculatus* (Poli, 1791) provide insights into their specialised roles in the biology of a chemosymbiotic bivalve

**DOI:** 10.1186/s12864-019-6177-0

**Published:** 2019-11-07

**Authors:** Benedict Yuen, Julia Polzin, Jillian M. Petersen

**Affiliations:** Centre for Microbiology and Environmental Systems Science, Division of Microbial Ecology, University of Vienna, Althanstrasse 14, 1090 Vienna, Austria

**Keywords:** Chemosynthetic symbiosis, RNA-Seq, Bivalve, Lucinid, Innate immunity, Nutritional symbiosis

## Abstract

**Background:**

The lucinid clam *Loripes orbiculatus* lives in a nutritional symbiosis with sulphur-oxidizing bacteria housed in its gills. Although our understanding of the lucinid endosymbiont physiology and metabolism has made significant progress, relatively little is known about how the host regulates the symbiosis at the genetic and molecular levels. We generated transcriptomes from four *L. orbiculatus* organs (gills, foot, visceral mass, and mantle) for differential expression analyses, to better understand this clam’s physiological adaptations to a chemosymbiotic lifestyle, and how it regulates nutritional and immune interactions with its symbionts.

**Results:**

The transcriptome profile of the symbiont-housing gill suggests the regulation of apoptosis and innate immunity are important processes in this organ. We also identified many transcripts encoding ion transporters from the solute carrier family that possibly allow metabolite exchange between host and symbiont. Despite the clam holobiont’s clear reliance on chemosynthesis, the clam’s visceral mass, which contains the digestive tract, is characterised by enzymes involved in digestion, carbohydrate recognition and metabolism, suggesting that *L. orbiculatus* has a mixotrophic diet. The foot transcriptome is dominated by the biosynthesis of glycoproteins for the construction of mucus tubes, and receptors that mediate the detection of chemical cues in the environment.

**Conclusions:**

The transcriptome profiles of gills, mantle, foot and visceral mass provide insights into the molecular basis underlying the functional specialisation of bivalve organs adapted to a chemosymbiotic lifestyle.

## Background

Associations between invertebrates and chemoautotrophic bacteria contributed to the evolutionary success of diverse animal lineages, and are also fundamental to the functioning of marine ecosystems [1, 2]. One prominent example is chemosynthetic symbioses that are found in at least seven phyla of metazoan invertebrates [2]. In the deep sea, these symbioses underpin primary productivity that supports an unexpectedly large biomass (hydrothermal vent mussels and tubeworms) in a food scarce environment, thus serving as the foundation of entire ecosystems [1, 2]. Invertebrates that host chemoautotrophic bacteria are also ubiquitous in shallow water habitats and lucinid clams, in particular, are highly abundant members of the macrofaunal community in shallow water sediments. Indeed, lucinids are the most species rich and widely distributed group of chemosynthetic bivalves [3], and their ecological and evolutionary success can be attributed to their highly specific association with a single phylotype of sulphur-oxidising gammaproteobacteria.

The symbiosis between lucinid clams and sulphur-oxidising bacteria is a nutritional strategy; the symbionts use reduced sulphur compounds – produced by organic matter decomposing in anoxic sediment – as electron donors and autotrophically fix carbon dioxide to synthesise organic compounds that are transferred to the host [1]. Lucinid clams possess multiple atypical morphological traits associated with the peculiar nature of this nutritional strategy. To house significant bacterial biomass, the gills of lucinids are modified into a plump and conspicuous symbiont-housing organ with a large surface area extending beyond the limits of the visceral mass [4, 5]. The gill symbionts are enclosed in vacuoles of specialised cells known as bacteriocytes and the gills can constitute up to a third of the host’s weight in soft tissue [6]. This heavy reliance on symbionts for nutrition is reflected in the morphological simplification of the lucinid feeding structures and digestive system; poorly developed labial palps, a truncated stomach and digestive glands with a reduced number of tubules [4]. The lucinid foot is also modified to become vermiform and highly extensible (up to 5 times body length) [4]. In addition to locomotory and burrowing activities, the foot is responsible for building a tube connecting the clam to the sediment-water interface, which is a functional replacement for the loss of their inhalant siphon [4]. The lucinid foot also constructs tubes burrowing into the sediments below the animal to mine pockets of dissolved sulphide associated with decaying organic matter or the insoluble iron sulphides attached to sediment, in order to support the sulphide-requiring metabolism of their endosymbionts [7]. These unusual morphological adaptations to a chemosymbiotic lifestyle have allowed lucinids to successfully exploit the deep layers of sulphide-rich sediment, an ecological niche inaccessible to most other animals.

A wealth of literature documents the taxonomy and ecology of lucinid symbioses [4, 8–10], and we are beginning to unravel metabolic and physiological capabilities of the endosymbionts [11–13]. However, we still have little understanding of how the host regulates symbiotic interactions at the molecular level. Previous studies suggest a high degree of control over symbionts in the gills, as lucinids appear to inhibit symbiont cell division and are able to selectively digest symbionts or reacquire the same strain from the environment [14–16]. These studies suggest lucinid-symbiont crosstalk is not limited to nutrient exchange and may be regulated by other processes including host immunity. Indeed, immunity has been implicated in regulating interactions between deep sea mussels and the endosymbiotic bacteria harboured in their gills [17–19]. Additionally, non-symbiotic lucinid organs can also influence lucinid-symbiont interactions by controlling the availability of essential resources required for metabolism. Examples include filter feeding and digestion in the digestive tract [20–22], and sulphide and oxygen acquisition by the foot [7]. These examples show that the functions and physiology of the non-symbiotic host organs also contribute to the functioning of the chemosynthetic holobiont. Therefore, transcriptional profiles of symbiotic and non-symbiotic organs will provide more complete insights into how the lucinid host regulates this symbiosis.

**Fig. 1:**
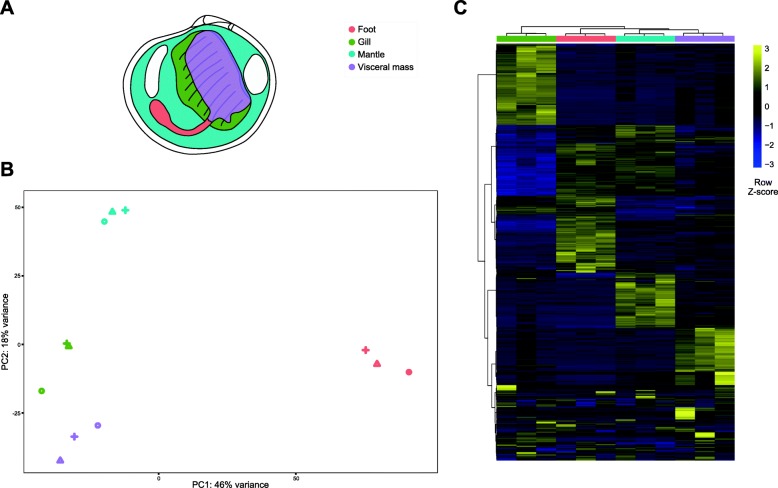
Overview of the differential gene expression experiment comparing transcriptomic profiles of four different *Loripes orbiculatus* organs. **a** Anatomical illustration of *L. orbiculatus*. Four different organs from three individuals were sampled for RNA-Seq: the gills, mantle, foot, and visceral mass. **b** Principal components analyses plotted using the transformed raw counts (variance stabilising transformation) of each sample, with PC1 on the horizontal axis and PC2 on the vertical axis. The samples from different individuals cluster together by organ type. Colours indicate the different organs and shapes indicate different clam specimens. **c** Expression patterns of genes (rows) specifically expressed in each organ (column). Yellow indicates high levels of expression while blue low indicates low levels of expression. The heatmap was plotted from VSD transformed counts and scaled by row. More details on the differentially expressed genes are in Additional file 3

We de novo assembled and annotated a reference transcriptome using RNA-Seq reads from four different organs (gills, mantle, visceral mass, and foot) of *Loripes orbiculatus* (Fig. 1 a), a lucinid clam widely distributed throughout the Mediterranean Sea. This served as the basis for differential gene expression analyses and the subsequent characterisation of organ-specific transcriptome profiles. We discuss the functional implications of organ-specific gene clusters, focusing on the symbiont-housing gills and the contribution of the non-symbiotic organs to regulating lucinid-symbiont interactions. These data are a valuable addition to the existing genomic resources for studying the lucinid symbiosis and have the potential to provide new insights into the molecular basis of animal-bacteria interactions.

## Results & discussion

### Assembly of a comprehensive lucinid transcriptome

We sequenced 12 RNA-Seq libraries from three replicates each of four organ types: the gills, foot, mantle, and visceral mass. After error correction, filtering, and trimming, each library comprised an average of 22,186,325 million read pairs that were de novo assembled into transcriptomes. These were combined with previously sequenced gill RNA-Seq libraries into a reference transcriptome of 104, 568 contigs with an N50 of 1365 bp, and 1996 contigs above 1 kbp in size (assembly statistics in Additional file 1). Analysis with BUSCO revealed 99% (97.1% complete and 1.8% partial) of the metazoan BUSCO gene set to be present; a higher level of completeness than most other published bivalve transcriptomes [23, 24]. This transcriptome is expected to be a valuable addition to the existing genetic resources for studying the molecular underpinnings of lucinid symbioses [11, 12]. Despite the large number of contigs in the final assembly, only 2.5% of the BUSCOs were identified as duplicates while 20,163 transcripts were functionally annotated with gene ontology terms. This could be due to a number of factors that might include a high frequency of uncharacterised lineage-specific genes and non-coding transcripts. Indeed, this large number of predicted gene models is not unprecedented as similar trends have been reported in other members of the Imparidentia; the draft genomes of *Ruditapes phillippinarum* and *Dreissena polymorpha* are predicted to encode over 100, 000 and 60, 000 genes, respectively [25, 26].

### Each organ is characterised by a unique transcriptome profile

We used this highly complete transcriptome as a reference to test for genes differentially expressed amongst the gill, visceral mass, mantle and foot (Fig. 1). Identifying clusters of organ-specific genes provides informative baseline data on the functions and physiology of each organ, and thus how both symbiotic and non-symbiotic organs may contribute to the functioning of the lucinid holobiont. A principal component analysis (PCA) of the variance stabilised dataset showed clear separation amongst the organs without any overlaps, indicating each organ has a clearly distinct transcriptomic profile (Fig. 1 b). This is supported by differential gene expression analyses, which showed that 1917, 1064, 823, and 999 corset unigenes were most highly expressed in the gill, visceral mass, mantle, and foot, respectively (Fig. 1 c). We then used gene set enrichment analyses of gene ontology (GO) terms and PFAM domains, as well as manual curation to gain deeper insights into the functional implications of these organ-specific gene clusters.

### Immunity and cell death are important processes in the symbiont-housing organ

To identify candidate immune genes involved in mediating lucinid clam-symbiont crosstalk, we used KEGG term annotation and conserved Interpro domains to analyse the reference transcriptome for metazoan immune pathways and receptors. The key components of the main immune signalling pathways conserved across most metazoan lineages were present in the transcriptome of *L. orbiculatus* (Additional file 2). We also investigated the repertoire of different pattern recognition receptor (PRR) gene families in the *L. orbiculatus* transcriptomes. PRRs of the evolutionarily ancient innate immune system detect Microbe-Associated Molecular Patterns, the non-self molecules that illicit the animal immune response [27, 28]. All major intracellular and extracellular PRR families typically observed in bivalve genomes are expressed by *L. orbiculatus* and we found no evidence massive immune gene family shrinkage nor loss of immune signalling pathways (Additional file 2). Some of these PRR families have undergone expansions similar to those in non-symbiotic bivalve genomes: the Toll-like receptors (TLRs), Scavenger Receptor Cysteine-Rich domain-containing genes (SRCRs), C1q-domain containing genes, and C-terminal fibrinogen-related domain-containing proteins (FReDs) (Additional file 2).

**Fig. 2:**
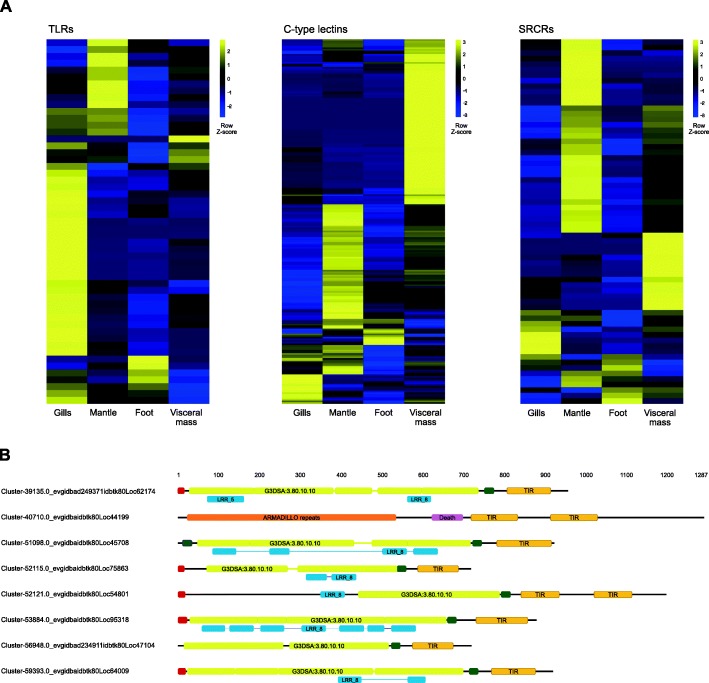
**a** Expression patterns genes from the Toll-like receptor, C-type lectin and Scavenger receptor cysteine-rich domain-containing protein pattern recognition receptor families (rows) across four *L. orbiculatus* organs (columns); G – gills, M – mantle, F – foot, V – visceral mass. Both differentially and non-differentially expressed unigenes were included for an overview of the expression profile of each family across the different organs. Yellow indicates high levels of expression while blue low indicates low levels of expression. The heatmap was plotted from VSD transformed counts and scaled by row. **b** Domain architectures of eight TIR domain-containing unigenes significantly more highly expressed specifically in the symbiont-housing gills. The numbers above indicate length in base pairs. Domains are depicted by coloured boxes; Bright yellow – G3DSA:3.80.10.10 (GENE3D leucine-rich repeat), darker yellow – TIR domain, blue – PFAM leucine rich repeats, pink – death, red – signal peptide, green – transmembrane domain

To gain insights into their functions, we analysed the expression patterns of these *L. orbiculatus* PRRs across the different organs and found that different PRR families have organ-specific expressions profiles. For example, more TIR domain containing unigenes (including TLRs) are more abundant in the gills than in other organs, while SRCRs and C-type lectins are more abundant in the mantle and visceral mass, respectively (Fig. 2 a). The organ specificity of these PRR families suggests that each organ represents a unique immunological environment with challenges specific to the physiology and function of each organ [29]. We then focussed on the symbiont-housing gills to identify candidate innate immune genes potentially involved in regulating lucinid-symbiont interactions. An array of unigenes encoding diverse PRRs are more abundant in the gills, including eight TIR domain-containing (PF01582) unigenes, four SRCRs, one Peptidoglycan Recognition Protein, and three C-type lectins (Additional file 3). Based on their conserved structural organisation, seven of the nine TIR domain-containing unigenes can be considered true TLRs (Fig. 2 b). One of these is a “twin-TIR TLR” marked by the presence of two intracellular TIR domains, an unusual structure previously reported only in other bivalves [30]. The conserved role of TLRs in mediating beneficial host-microbe interactions across metazoan lineages [27], in conjunction with their potential to be localised on endosomal membranes, makes the TLRs excellent candidates for mediating lucinid-symbiont crosstalk.

TLRs recruit proteins downstream to transduce their signals through the dimerization of the cytosolic TIR domain with the TIR domain of adaptor proteins [31]. The remaining TIR domain-containing unigene highly expressed in the gills is unlikely to mediate MAMP recognition because it lacks the conserved organisation of TLRs and is further predicted to be localised to the cytosol as evidenced by the absence of a transmembrane domain or signal peptide motif (Fig. 2 b). However, it could regulate lucinid-symbiont interactions by transducing signals from TLRs through their TIR domains. This gene comprises a long N-terminal region containing ARMADILLO repeat-like motifs, a central Death domain, and two N-terminal TIR domains (Fig. 2 b); an unusual domain composition characteristic of the ecTIR-DC family 6 genes reported only in other invertebrates [30]. The Death and TIR domain combination is characteristic of MyD88, the adaptor protein that transduces signals from ligand-bound TLRs [31], while ARMADILLO repeats are also found on proteins that regulate immune signalling [32]. The functions of these domains and the co-expression of this unigene with TLRs in the gills leads us to speculate it could be involved in transducing TLR signals.

**Fig. 3:**
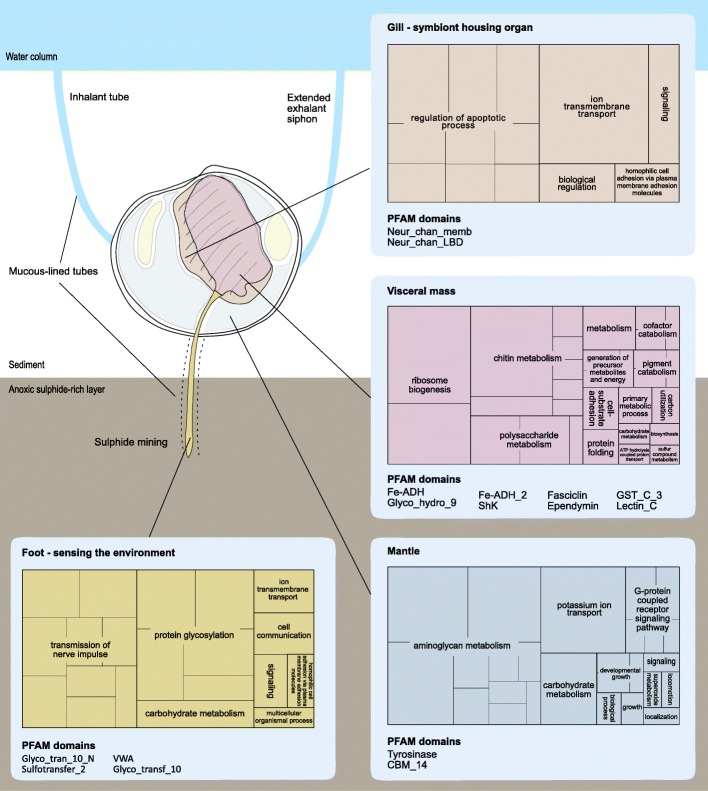
The Biological Process GO terms and PFAM domains enriched in organ-specific gene clusters reflect the unique functions of each organ and the roles they play in lucinid biology. The treemaps indicate non-redundant GO terms statistically enriched amongst the genes specifically upregulated in each organ. Each section within a treemap corresponds to a non-redundant GO term and the sizes of the boxes represent the log10 transformed adjusted *p* values from the GO term enrichment analysis. The treemaps were constructed using the REVIGO software and GO terms were clustered using the SimRel measure at a threshold > 0.7 similarity. The statistically enriched PFAM domains in each organ-specific gene cluster are indicated below the treemaps

**Fig. 4:**
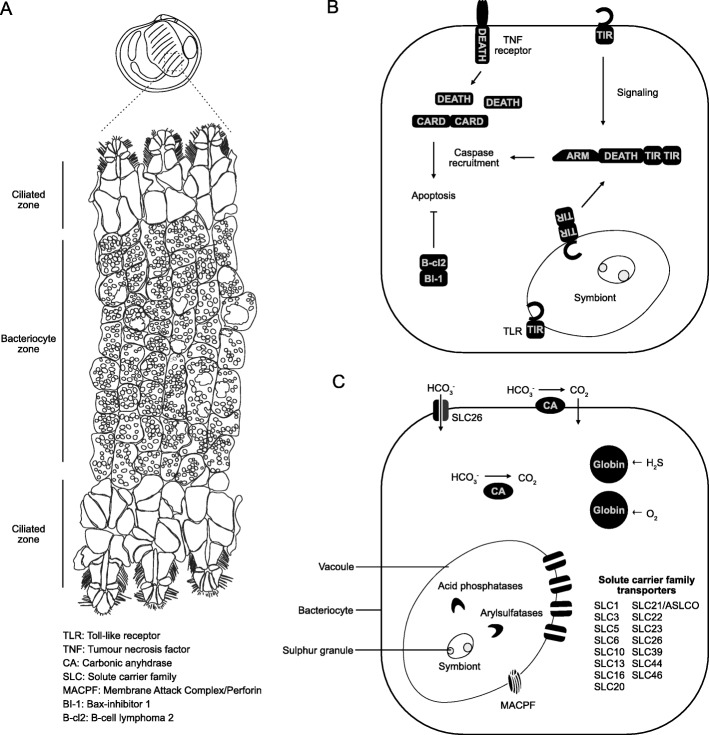
Genes expressed specifically in the symbiont-housing gills of *Loripes orbiculatus* and putatively involved in symbiosis. **a** The organisation of gill filaments along the transverse plane. The *L. orbiculatus* gill filaments are organised into a ciliated zone that is devoid of symbionts and a bacteriocyte zone. **b** The receptors and molecular signalling pathways involved in innate immunity and apoptosis. These molecular pathways could regulate apoptosis in either the ciliated zone or the bacteriocyte zone. DEATH – death domain (PF00531); CARD – card domain (PF00619); TIR – Tir domain (PF01582 or PF13676); ARM – armadillo repeat region (PF00514). **c** Metabolite transport, intracellular digestion, facilitation of symbiont chemoautotrophic metabolism. Pointed arrows indicate putative direction of pathway activity. Bar-ending arrows indicate inhibitory activity

Biological Process (BP) GO terms associated with apoptosis regulation are enriched amongst the unigenes expressed in the gills (Fig. 3). One of these encodes a tumour necrosis factor receptor (TNFR) with an intracellular Death domain (Fig. 4), which is a structure typical of the type I TNFRs responsible for activating apoptosis and inflammatory signalling in mammals [33]. However, the majority of these apoptosis-related unigenes simply contain CARD (PF00619) or Death domains (PF00531), which are usually associated with adaptor proteins that mediate cell death signalling by recruiting caspases through homotypic interactions [34]. The importance of regulating apoptosis in the gills is further emphasised by the expression of unigenes encoding B-cl2-like and Bax-inhibitor 1-like proteins that supress cell death (Fig. 4, Additional file 3) [35, 36]. It is noteworthy that an expanded repertoire of Death domain-containing genes was reported in the genome of *Bathymodiolus platifrons*, a deep sea mussel that also hosts chemoautotrophic endosymbionts [19]. Interestingly, TUNEL staining of the gill tissues of lucinid and chemosynthetic deep sea mytilids indicates cell death is prevalent in the ciliated zone of both lucinid and symbiotic deep sea mytilids but not in the bacteriocyte zone [37, 38]. These lines of evidence suggest that apoptosis regulation is a key process in the symbiont housing organs of chemosynthetic bivalves in general. High rates of chemoautotrophic metabolism imposes an oxygen demand upon animal hosts that is typically much higher than for its non-symbiotic relatives [39]. Therefore, one possible explanation is that the high prevalence of apoptosis in ciliated cells and enrichment of apoptosis-related GO terms in the lucinid gills are due to a high cell turnover rate caused by greater oxidative stress. However, recent work has shown that genes involved in regulating apoptosis are more highly expressed in bathymodiolin mussels when their symbiont load is experimentally depleted [40], which suggests the role of apoptosis in chemosynthetic symbioses may be more complicated.

The expression of these diverse molecules involved in various arms of innate immunity suggests cells in the lucinid gills have the capacity to detect and raise a complex response to bacteria. We cannot rule out the possibility that the PRRs, immune signalling and effector molecules expressed in the gills are used to defend the host against harmful bacteria because the gills are constantly exposed to the water from the environment. An important caveat to note is that the KEGG immune signalling pathway annotations are biased towards classical model organisms and little conclusive functional evidence exists to support the involvement of proteins containing the domains discussed (e.g. C-type lectin, Death and SRCR) in bivalve immunity. Nevertheless, these data allow to shortlist candidates for future studies investigating the role of innate immunity in lucinid-symbiont crosstalk as well as candidates for investigating the roles of cell death in regulating endosymbiont abundance or bacteriocyte turnover dynamics.

### Unigenes expressed in the gill facilitate the chemoautotrophic metabolism of the endosymbiotic bacteria

The beating of the lateral cilia on bivalve gills generates the water currents that facilitate gas exchange and filter-feeding, and previous studies indicate this activity is under neurohormonal control [41]. This is consistent with the enrichment of BP GO terms associated with ion transmembrane transport in the gills, because half of the unigenes contributing to this GO term encode ion channels typical of the animal nervous system known as nicotinic acetylcholine receptors (Fig. 3; Additional file 3). Unigenes associated with the dynein complex are also enriched amongst the cellular component GO terms (Additional file 4); dyneins are critical to ciliary movement because they convert ATP to mechanical motion [42]. Nicotinic acetylcholine receptors and dynein complex genes are similarly over-represented in the gills of non-chemosymbiotic bivalves [23], which indicates that these genes are unlikely to be directly involved in the unique symbiont housing role of lucinid gills. Nevertheless, their functions influence host-symbiont interactions because the water flow driven by ciliary beating ensures symbionts are supplied with the oxygen and sulphide necessary for chemoautotrophic metabolism.

In order to supply their symbionts with resources for chemoautotrophic metabolism, the haemoglobins of thioautotrophic invertebrates have evolved to bind sulphide and oxygen while preventing the rapid oxidation of sulphides [39]. Two globin domain-containing unigenes are expressed in the gills, one of which groups with the sulphide-binding hemoglobin HBI of the lucinid *Phacoides pectinatus*, while the other forms a clade with *P. pectinatus* HBII and HBIII, which are responsible for oxygen binding (Additional file 5). These findings suggest that like other invertebrates associated with thioautrophic symbionts, *L. orbiculatus* uses specialised haemoglobins to supply its symbionts with sulphide and oxygen. Through the oxidation of sulphur, endosymbionts produce energy that is used to fixed inorganic carbon in the form of CO_2_ into sugars [1]. However, dissolved CO_2_ levels are generally low in seawater and the spontaneous generation of CO_2_ from HCO_3_^−^ is minimal due to the high alkalinity of sea water. To solve this physiological challenge, all known thioautotrophic symbioses rely on carbonic anhydrases (CAs) to catalyse the reversible dehydration of bicarbonate to carbon dioxide, which diffuses easily across cell membranes and facilitates inorganic carbon uptake [43]. Two CA unigenes are expressed in the gills (Fig. 4, Additional file 3, Additional file 4), both of which have signal peptides that indicate they are secreted. However, the larger of the two has a predicted GPI anchor site (Serine position 432) that could mediate the attachment of the protein to the cell membrane (Additional file 5). A similar secreted CA bearing a GPI anchor site is expressed in bacteriocytes of the chemosynthetic vesicomyid clam *Phreagena okutanii* and is probably responsible for catalysing the conversion of HCO_3_^−^ in the hemolymph into CO_2_ that can diffuse into the bacteriocytes [44]. The CAs in bivalves regulate multiple physiological functions including respiration, calcification and mineralization [45, 46]. However, in addition to these other physiological roles, we hypothesise that the CAs expressed in the gills of *L. orbiculatus* could be localised to the bacteriocyte cell membrane or secreted into the gill hemolymph to make inorganic carbon available of to the endosymbionts. An alternative but non-mutually exclusive route for providing the symbionts with inorganic carbon is to transport HCO_3_^−^ directly into the bacteriocytes. Diatoms rely on the SLC4 family of carbonate anion transporters to obtain the inorganic carbon required for photosynthesis [47]. Unigenes encoding SLC4 were not more abundant in the gills compared to the other organs analysed, but we identified a unigene from the SLC26 family of transporters more highly expressed in the gills (Fig. 4, Additional file 4, Additional file 5). This SLC26 unigene has a highly conserved domain architecture and forms a clade with *Drosophila melanogaster* and *Anopheles gambiae* genes encoding Prestin (Additional file 5), a protein with the capacity to transport bicarbonate, oxalate, and sulphate anions [48]. The role of SLC26 in transporting bicarbonate into the bacteriocytes is worth further investigation as the symbiont genome encodes multiple carbonic anhydrases that could convert bicarbonate into CO_2_ in bacteriocytes [12].

### The lucinid metabolism relies on both nutrient translocation and heterotrophy

Previous studies have inferred that lucinids acquire nutrients from their symbionts through a carbon transfer strategy known as “milking”, whereby nutrients are ‘leaked’ or translocated to the host following carbon fixation [49]. This was supported by microscopic evidence indicating low rates of symbiont digestion in the gills and that symbiont cell division is inhibited [15, 50]. Expressed in the gills are 20 unigenes encoding transporters belonging the to the Solute Carrier (SLC), and folate (Additional file 5). Indeed, the SLC family has been implicated in regulating nutrient transfer in family that contribute to the enrichment of BP GO terms associated with ion transmembrane transport (Fig. 4, Additional file 5). There are a total 46 SLC families that are united by a common mode of function – they use the ion gradients across cell membranes to drive the transmembrane transport of solutes. Fifteen different SLC families are expressed in the gills, with specificities for a diversity of substrates including amino acids, glucose, phosphate, monocarboxylates, organic cations and anions, zinc, ascorbic acidother nutritional symbioses. The SLC5 and SLC46A3 families are thought to regulate nutrient transfer between cnidarians and endosymbiotic algae [51, 52], while members of SLC families 5, 6, 13, and 16 are enriched in the symbiont-housing root of the bone eating worm *Osedax japonicas* [53]. The diverse transporters expressed in the *L. orbiculatus* gill therefore likely facilitate lucinid-endosymbiont nutrient exchange and supports the inference that the host obtains nutrients through “milking”, although intracellular digestion may also play a role (see below).

We also identified unigenes from other transporter superfamilies in the gills, including monocarboxylate and organic cation transporters from the Major Facilitator Superfamily, and a DUR3-like unigene encoding a urea-proton symporter (Fig. 4, Additional file 5). DUR3 is thought to be involved in nitrogen transfer between invertebrate hosts (Hydra and the giant clam) and their algal symbionts [54, 55], but the expression of a urea transporter in the lucinid gills is somewhat unexpected because *Candidatus* Thiodiazotropha endoloripes, the endosymbiont of *L. orbiculatus*, has the capacity to fix nitrogen. Nevertheless, the endosymbiont genome also encodes the transporters and enzymes (Additional file 5) necessary for metabolising urea [12], which suggests the *L. orbiculatus* DUR3 could be used to provide symbionts with a nitrogen source in the form of urea. The recycling of nitrogenous compounds like urea could be a symbiont strategy for coping with nitrogen-limitation while also helping the host remove metabolic waste. An alternative possibility is that the lucinid host could play an important role in controlling symbiont nitrogen metabolism. Indeed, the pea plant prevents rhizobium from assimilating ammonium by providing the bacteria with amino acids, thereby regulating symbiotic nitrogen fixation [56]. It is thus interesting to speculate that metabolite transporters could also serve as a mechanism for controlling symbionts by regulating access to nutrients.

Metabolic flexibility in both *L. orbiculatus* and its symbionts is likely important in the coastal sediments of Elba where the waters are oligotrophic and the availability of sulphide fluctuates in time and space [57, 58]. Biochemical signals of acid phosphatase and arylsulfatase activity, both markers of lysosomal activity, were detected in freshly collected clams indicating that lucinids also directly digest their symbionts [50]. This is consistent with the expression of unigenes encoding proteins involved in lysosomal activity in the gills, including two acid phosphatases, two arylsulfatases, and one Cathepsin L (Fig. 4, Additional file 3). We also identified two unigenes from the Membrane Attack Complex/Perforin (MACPF) superfamily (PF01823) expressed in the gills. Proteins from this superfamily are pore forming toxins involved in immunity that form holes in the membrane of bacteria leading to cell lysis [59]. A MACPF gene in the oyster *Crassostrea gigas* is upregulated under bacterial exposure, localised to late endosomes, and is highly expressed in the gills, digestive gland and gonads [60]. While it is possible the lucinid MACPF proteins are involved primarily in immune defence, these genes could potentially be co-opted to facilitate the digestion of endosymbiont bacteria.

Gill endosymbiont digestion is, however, unlikely to play a significant role in nutrient acquisition except under exceptional circumstances such as prolonged sulphide limitation [50]. In fact, many more enzymes typically associated with metazoan digestion are highly expressed in the lucinid visceral mass rather than the gills (Additional file 3). This includes carboxypeptidases, papain and pepsin family aspartic peptidases such as cathepsins (D, F, and L), as well as chymotrypsin and subtilisin family serine peptidases (Additional file 3). The importance of digestion in the visceral mass is further reflected in the enrichment of BP GO terms with chitin and polysaccharide metabolism, as well as the Glycosyl hydrolase family 9 PFAM domain (PF00759) (Fig. 3). These expression patterns suggest the lucinid digestive system is able to digest complex polysaccharides that are typical constituents of phytoplankton in the marine environment [61]. This correlates with the enrichment of lectin C-type domains (PF00059) – corresponding to 25 C-type lectin unigenes – in the visceral mass, suggesting that carbohydrate recognition is important in the lucinid gut. C-type lectins are characterised by having one or more copies of the C-type lectin domain, and they fulfil diverse immunological and physiological roles through the recognition of an array of primarily sugar-based ligands [62]. In the mosquito gut, C-type lectins bind to microbes to enable antimicrobial peptide evasion, thereby allowing microbial colonisation and gut homeostasis [63]. It is likely that the lucinid digestive track is also associated with a thus far unstudied microbiota, and the visceral mass C-type lectins could thus play a role in gut immunity and homeostasis. Alternatively, the lucinid C-type lectins could also regulate food sorting as in *Crassostrea gigas*, where they are expressed on the mucosal surfaces of feeding organs [64]. Regardless of their precise roles, these lines of evidence suggest that heterotrophy remains an important strategy for acquiring nutrients despite the morphological simplification of the lucinid digestive system and its symbiosis with chemosynthetic bacteria. Our findings thus provide molecular support for previous observations that lucinids are able to capture and ingest particulate organic matter [20].

### The foot as a sensor and regulator of interactions with the environment

The BP GO terms enriched in the foot include transmission of nerve impulse, protein glycosylation and carbohydrate metabolism (Fig. 3). These GO terms correlate with the enrichment of Glycosyl transferase (PF17039 and PF00852), sulfotransferase (PF03567), and von wildebrand A (PF00092) PFAM domains in the foot (Fig. 3). VWA domains are typically found in glycoproteins that form the basis of mucus and are secreted into the extracellular matrix [65, 66]. These proteins are often post-translationally modified with glycan attachment in a process known as glycosylation involving enzymes called glycosyltransferases. Further post-translational modifications are carried out by various enzymes such as sulfotransferases and fucosyltransferases [67]. Therefore, the enrichment of GO terms and PFAM domains associated with glycosylation indicates a battery of genes expressed in the lucinid foot are dedicated to mucus biosynthesis and is consistent with the foot’s ecological and physiological functions. Unlike most other bivalves, lucinids use the foot to build a mucus-lined inhalant tube to access the oxygenated water column while they inhabit the deeper sulphide-rich sediment layers inaccessible to other bivalves [4]. They also dig mucus-lined sulphide-mining tubes ventrally to probe deeper sediments for sulphide to provide to their symbionts [5, 7]. By contrast, gene expression in the non-symbiotic mussel foot is instead dominated by byssal cuticle proteins and other proteins that contribute to connective tissue structures [23], which reflects the mussel foot’s primary role in secreting byssal threads that facilitate attachment to hard substrates.

The chemosynthetic invertebrates inhabiting deep sea hydrothermal vent ecosystems are constantly bathed in dissolved sulphide, while lucinids, on the other hand, inhabit sediments where sulphides levels may be low or patchy [5, 7]. Consequently, the lucinid foot has become a specialised probe for locating and acquiring sulphide in the clam’s surrounding environment [5, 7]. The enrichment of GO terms associated with nerve impulse transmission indicates that the lucinid nervous system plays an important role in coordinating this behaviour (Fig. 3); unigenes contributing to this GO term include both nicotinic acetylcholine receptors and ionotropic glutamate receptors (iGluRs). In muscles, the former are located at neuromuscular junctions where electrical signals from neurons control the contraction of muscle cells [68]. Three iGluRs unigenes are expressed in the foot, one of which belongs to the NMDA2 subfamily while the remaining two are members of the AKDF subfamily (Additional file 3). iGluRs similarly mediate synaptic transmission throughout the nervous system, and while they do not directly bind H_2_S, it is interesting that NMDA2 receptor signalling is enhanced by physiological levels of H_2_S in the mammalian nervous system [69, 70]. Although little is known about the receptors specific for H_2_S, it is interesting to speculate that the *iGluRs* expressed in the foot could be involved in coordinating the clam’s behavioural response to sulphide in the environment.

G-protein coupled receptors (GPCRs) are also more abundant in the foot (16 unigenes) than in all other organs, except the mantle (18 unigenes), which is consistent with the mantle’s role in sensing and initiating responses to environmental cues [71]. Based on the Interpro annotations of the unigenes, most of the GPCRs expressed in the foot contain a 7tm_1 domain (PF0001) and have short N-termini regions, which indicate that they belong to the rhodopsin family of GPCRs [72]. GPCRs belonging to this family interact with a broad spectrum of ligands and their expression in the lucinid foot suggests they could play a role in enabling the foot to detect cues in the immediate environment. Furthermore, the deployment of non-overlapping subsets of GPCRs in the mantle and foot also suggests the molecular set-up for sensing environmental cues is specialised to the distinct functions of each organ (Additional file 3). In addition to receptors for chemical stimuli, a unigene encoding a piezo-type mechanosensitive ion channel component is also expressed in the foot; this unigene is likely to be important for mechanical signal transduction as the foot probes the sediment [73]. These findings indicate the lucinid foot possesses an intricate array of receptors deployed for probing the environment and we hypothesise these chemosensory abilities play an important role in detecting and acquiring resources for the symbiosis.

## Conclusions

The global transcriptomic profiles of the *L. orbiculatus* organs are broadly consistent with the expected functions of each organ based on our current understanding of lucinid biology and ecology. However, our findings also allow us to generate new questions to address in future studies. For example, in addition to detecting resources in the environment, does the foot also play a role in replenishing symbiont abundance by secreting mucus that serves as a chemoattractant and first point of contact a potential symbiont might have with the host? Given the importance of heterotrophy, how do seasonal changes to resource availability in the environment (sediment sulphide and phytoplankton abundance) influence the dynamics of nutritional and immune exchanges in the symbiont-housing gills? The transcriptome we have assembled and the accompanying insights it has provided into molecular basis each lucinid organ’s functions, set up the foundation for future studies to build a picture of how lucinids use their molecular toolkit to orchestrate the tripartite interactions linking host, symbiont and the environment.

## Methods

### Sample collection and processing

*L. orbiculatus* (roughly 1.5 cm in length) were dug up from 10 to 20 cm deep sediment in the Bay of Fetovaia, Elba, Italy (May 2017). The clams were brought back to the Hydra Institute where they were kept in sediment and aerated sea water from the bay for no longer than 3 h before they were fixed in RNAlater (Thermo Fisher Scientific, Vilnius, Lithuania) and stored at − 20 °C. The gills, mantle, visceral mass, and foot from three individuals were dissected, placed in a tube containing sterile glass beads and TRIzol (Thermo Fisher Scientific), and subsequently homogenised using a pellet pestle. Total RNA was extracted following the manufacturer’s instructions. The extracted RNA were quantified using a Qubit 3.0 fluorometer (Thermo Fisher Scientific) and their quality assessed using the RNA 6000 Nano Kit on the Agilent 2100 Bioanalyzer (Agilent Technologies, Santa Clara, USA).

To generate a comprehensive reference assembly, we also included 5 additional gill cDNA libraries that were previously sequenced by the group. These gill samples were excised from freshly collected specimens from the same location Elba in October 2015 and from the same batch of specimens that were brought back to the University of Vienna and kept in oxygenated water and Elba sediment until December 2016. The samples were fixed and RNA was extracted as described above. Each RNA extract from these samples was DNase-treated with the Turbo DNA-free Kit (Thermo Fisher Scientific, Vilnius, Lithuania) following the manufacturer’s instructions. The treated RNA was ethanol precipitated by adding 150 μl DEPC water, 20 μl sodium acetate (3 M, pH 5.2), 2 μl glycogen (20 mg/ml), 660 μl ethanol and precipitated at − 80 °C for 30 min. The precipitated RNA was then pelleted at 21,000 x g for 15 min at 4 °C, washed with 70% ethanol, centrifuged at 21,000 x g for 10 min at 4 °C, air dried, and eluted in 30 μl DEPC water.

### Library construction and sequencing, sequence pre-processing, and filtering

The RNA samples were poly (A) enriched and cDNA libraries were prepared using the NEBNext® Ultra™ RNA Library Prep Kit for Illumina (New England Biolabs, Ipswich, MA) by the Vienna Biocenter Core Facilities GmbH (VBCF, Vienna, Austria). A total of 12 stranded RNA-Seq libraries (4 tissues × 3 biological replicates per tissue) were multiplexed and sequenced with TruSeq V4 chemistry by the VBCF in one lane of a flow cell on the Illumina HiSeq 2500 to generate 125 bp paired-end reads. Predicted errors in the raw reads were corrected using Rcorrector under default settings and the uncorrectable reads were removed [74]. SortMeRNA was used to remove rRNA sequences from the libraries by searching against the 5S, 5.8S, 16S, 23S, 18S, and 28S rRNA sequences in the package’s default rRNA database [75]. The filtered and corrected reads were then trimmed using Trimmomatic v0.36 with the following settings “SLIDINGWINDOW:4:15 MINLEN:36” [76]. Illumina adapter sequences were also removed from the paired-end libraries with the option ILLUMINACLIP:adapters/TruSeq3-PE-2.fa:2:30. The quality of the trimmed and filtered reads were assessed using FastQC v0.11.5, taking into consideration per base quality scores, GC-content, and read length (http://www.bioinformatics.bbsrc.ac.uk/projects/fastqc).

For the additional 5 gill libraries that were added to our comprehensive reference dataset, the RNA samples were processed with a ribosomal RNA depletion step using the Ribo-Zero Gold rRNA removal Kit (Epidemiology) prior to library preparation. Library preparation and sequencing were then carried out by the VBCF as described above. These libraries were processed using the same workflow as the other libraries sequenced for the present study but with an additional step to remove symbiont-derived reads. Briefly, after filtering out rRNA reads using SortMeRNA, the remaining reads were mapped under default parameters to the symbiont genome (accession IDs LVJW00000000 and LVKA00000000; genomes available on NCBI in in BioProject PRJNA314435), using Bowtie2 v2.3.3.1 [12, 77]. The unmapped reads were retained for trimming and processing as described above.

### De novo transcriptome assembly, quality assessment

Each filtered library of reads was individually assembled into contiguous cDNA sequences with IDBA_tran v1.1.3 with the parameters -max_isoforms 100 --mink 20 --maxk 80 --step 5 [78]. The assembled libraries were then concatenated and CD-HIT-EST v4.7 was used to cluster the contigs and stringently remove redundancies with the parameters -c 1.0 -G 0 -aS 1.0 -aL 0.005 [79]. The Evidential Gene tr2aacds pipeline was used to predict the coding sequence regions within the transcripts (https://sourceforge.net/projects/evidentialgene/), and highly similar peptide sequences (90% identity) were clustered using CD-HIT v4.7 (−c 0.90) to create the final list of transcripts [79]. We used TransRate to assess the assembled transcriptome with variety of metrics, including number of transcripts, total number of bases, N50 values and GC content using [80]. Assembled transcripts with predicted open reading frames shorter than 80 amino acids were filtered out. Although small open reading frames and other non-coding transcripts are likely to have important albeit unknown biological functions, this arbitrary threshold was chosen to reduce the number of transcripts in the final assembly to reduce the computational resources required for subsequent analyses. We used the BUSCO program v3.0.2 to assess the completeness of the final reference transcriptome through a search against a set of 978 metazoan BUSCOs (Benchmarking set of Universal Single-Copy Orthologs) [81, 82].

### Functional annotation of the predicted proteins

The predicted peptide sequences were annotated with protein domain and conserved motif information using InterProScan (5.28–67.0) [83] and hmmscan against the PFAM-A database [84]. We also annotated the predicted proteins by blastp searching against the SwissProt database (−max_target_seqs 1 -evalue 1e-6). The BLAST and InterProScan outputs were combined and then annotated with GO terms using BLAST2GO v5.0.21 [85, 86]. The predicted peptide sequences were also annotated with KEGG terms using the BlastKOALA webserver (https://www.kegg.jp/blastkoala/) and then mapped to the KEGG pathways database to identify unigenes involved in immune signalling [87, 88].

### Identification of differentially expressed genes and functional enrichment of organ-specific expression profiles

Only the libraries sequenced from the organs collected in May 2017 were used for differential expression analyses. We used a combination of Salmon and Corset to map reads to each transcript and cluster transcripts into putative unigenes [89, 90]; each unigene is assembled from transcripts that appear to originate from the same transcriptional locus. A reference transcriptome index was constructed using Salmon v0.9.1 with the parameters “--type quasi -k 31”, and we used the “pseudo-alignment” algorithm to map the reads from each library to the index with default settings [89]. The numbers of reads mapped to each transcript were generated using Corset v1.07 under default parameters but with ‘-m 0’ applied to retain all transcripts [90]. Corset uses the Fragment equivalence classes output by Salmon to cluster transcripts into putative genes based on sequence similarity and read mapping [90]. A Principal component analysis (PCA) of the vsd-transformed (variance stabilizing transformation) counts was used to explore the global transcriptomic variation across samples; implemented through DESeq2 version v1.18.1 according to instructions in the DESeq2 manual [91] and visualised using the R package “ggplot2” [92].

Analyses to identify unigenes differentially expressed across the organs were conducted through pairwise comparisons between each organ using DESeq2 v1.18.1 [91]. TXimport v1.6.0 was used to import the read count data from the Salmon output [93]. We excluded unigenes with fewer than 10 reads mapped across all samples to reduce computational demands. A false discovery rate (FDR) of 10% was implemented using the Benjamini–Hochberg method. An FDR-adjusted *p*-value ≤0.01 and log2 fold change threshold of 1 (equates to a minimum 2-fold change) were used to produce the final list of differentially expressed genes. Organ-specific clusters of unigenes upregulated in one organ versus all others were identified with a Venn diagram (http://bioinfogp.cnb.csic.es/tools/venny).

A hypergeometric test, implemented through the Cytoscape plug-in BiNGO v3.0.3, was used to test for statistical enrichment (FDR-adjusted *p*-value < 0.05) of GO terms in the lists of organ-specific unigenes [94]. The GO terms enriched in each organ were visually summarised using Revigo [95]. GO terms were clustered using semantic similarity measure (SimRel) in REVIGO and GO terms with > 0.7 redundancy were collapsed [95]. We also tested for statistically enriched PFAM domains amongst the different organs using a hypergeometric test and the *p*-values were adjusted for an FDR of 10%. The PFAM domain enrichment analyses were carried out in R using the script provided in Supplemental Text 1 of Chandran et al. (2009) [96].

## Supplementary information

**Additional file 1.** Assembly statistics – Assembly statistics for reference transcriptome

**Additional file 2. **Genes involved in immunity – Tables summarising the genes putatively involved in *L. orbiculatus* immunity

**Additional file 3.** Differentially expressed genes lists – Lists of genes differentially expressed in pairwise comparisons of the four organs analysed in this study

**Additional file 4.** GO term enrichments – Results of the gene ontology term enrichment analyses of genes highly expressed in each organ

**Additional file 5. **Trees and tables – Phylogenetic trees, table of the transporter genes annotated in the *L. orbiculatus* transcriptome, and table of the urease metabolism genes in the genome of *Loripes orbiculatus* endosymbiont *candidatus* Thiodiazotropha endoloripes

## Data Availability

Raw sequencing reads have been deposited in the NCBI SRA database under the umbrella Bioproject PRJNA555495, linked to Biosamples SAMN12321431–42. The final set of high quality assembled contigs are available from the corresponding author on request. Transcript annotation and expression data are available in Additional file 2, Additional file 3, and Additional file 5. The complete results of hypergeometric tests on organ-specific gene clusters are available in Additional file 4. Multiple sequence alignments for trees in Additional file 5 are available from the corresponding author on request.

## Abbreviations

ATP: Adenosine triphosphate
BP: Biological Process
BUSCO: Benchmarking Universal Single-Copy Ortholog
CA: Carbonic anhydrase
DEPC: Diethyl pyrocarbonate
FDR: False discovery rate
FReD: C-terminal fibrinogen-related domain
GO: Gene ontology
GPCR: G protein-coupled receptor
GPI: Glycosylphosphatidylinositol
iGluR: Ionotropic glutamate receptor
MACPF: Membrane attack complex/Perforin
MAMP: Microbe Associated Molecular Pattern
PCA: Principal component analysis
PRR: Pattern Recognition Receptor
RNA: Ribonucleic acid
SLC: Solute carrier
SRCR: Scavenger receptor cysteine-rich
TIR: Toll/interleukin-1 receptor
TLR: Toll-like receptor
TNFR: Tumor necrosis factor receptor
