# Supplementary material for: Organ transcriptomes of the lucinid clam Loripes orbiculatus (Poli, 1791) provide insights into their specialised roles in the biology of a chemosymbiotic bivalve
Source: BMC Genomics. 2019 Nov 7;20:820. doi: 10.1186/s12864-019-6177-0 (PMC6836662; doi:10.1186/s12864-019-6177-0)

**Globin ML tree (10000 bootstraps)**

Maximum likelihood tree constructed using IQ-TREE with globin peptide sequences retrieved from NCBI. Peptide sequences were aligned using MAFT and model of evolution was select through the in-built test implemented in IQ-TREE. SH-aLRT /ultrafast bootstrap support values are indicated on the branches.


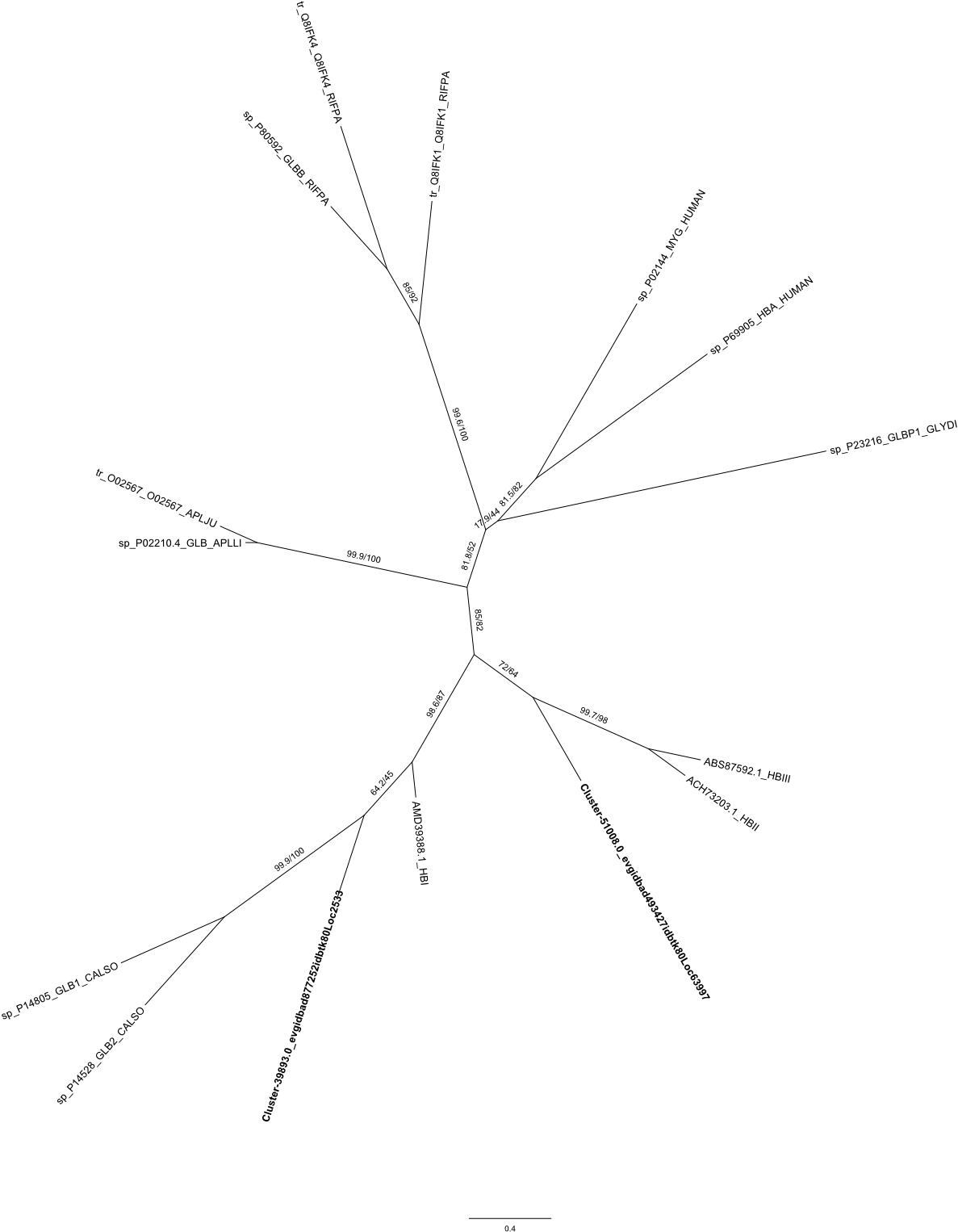


**Carbonic anhydrase GPI anchor motif prediction**

>Cluster-40745.0_evgidbad23029idbtk80Loc25335 | FPrate:0.000 | OMEGA:S-432

MLGRIVLFLGLVCLSDESGWGYVGEKGPANWDNLFPEACSGRYQSPIDINTSRTIYNPQLKDFAIFFDPP

SPGSKFYVHNNGHAVQVDTEGKFYVSNGGLPNIYSTAQFHFHWGHKSHHGSEHTIDGKAAPIEMHIVNWN

SDKFSSIAEAATEPEGLAVLGVLFEISREDNPVLEPIVQVLLDVRDPDMKIKAEIPAQSMRAFLPPAPEW

YYRYSGSLTTPKCFESVIWTVFKERQTISRRQLHVFRQVLKPKHHKKRHIKPSRAERDVLQELGIMDNVV

EKARLRRNLENKMQADSAAAKETVVLNEPDVTETNSSAEVTKYETHGTPNEAAGQGNTEHSNTKDKGSEH

GGHSMPKQDQSSNHGNAMRDPTVLVESNDREVFRHSLVNNYRPVQPLNGRTVYRSFPFFDTPIPSRSRSR

SKMDSVSVKGPSGGSSTIQISILTLAFGLLSALS

SLC26/Prestin tree (10000 bootstraps)

Maximum likelihood tree constructed using IQ-TREE with sequences analysed in Hirata et al (2012). Peptide sequences were aligned using MAFT and model of evolution was select through the in-built test implemented in IQ-TREE. SH-aLRT /ultrafast bootstrap support values are indicated on the branches.

Hirata T, Czapar A, Brin L, Haritonova A, Bondeson DP, Linser P, Cabrero P, Thompson J, Dow JA, Romero MF: **Ion and solute transport by Prestin in *Drosophila* and *Anopheles***. *Journal of insect physiology* 2012, **58**(4):563-569.


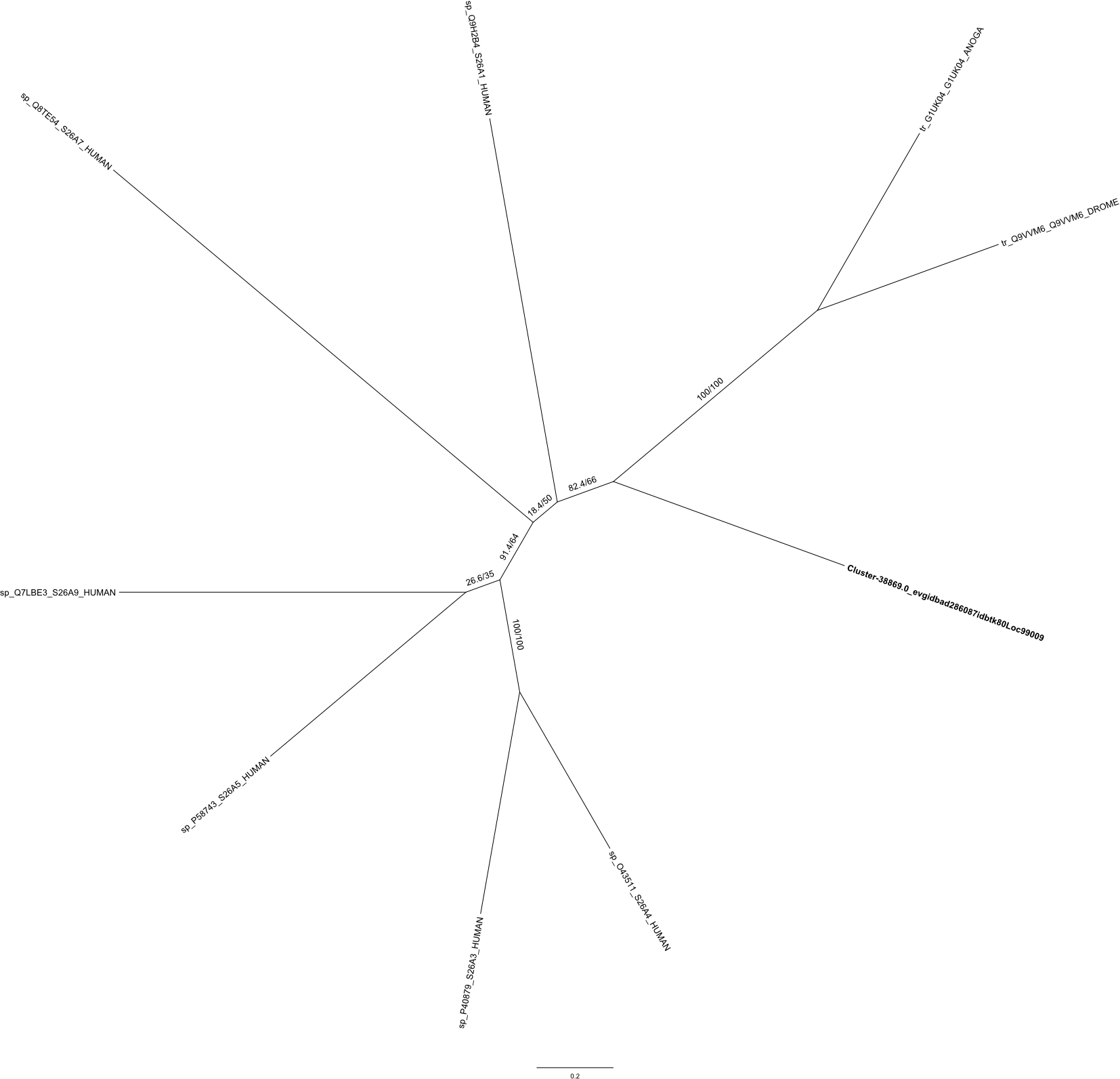


**Solute carrier family and other transporters**

| **Transporter** | KEGG ID |  |  |  |  |  |  |  |  |  |
| --- | --- | --- | --- | --- | --- | --- | --- | --- | --- | --- |
| **ABC Transporters, Eukaryotic Type** |  |  |  |  |  |  |  |  |  |  |
| ABCC (CFTR/MRP) subfamily |  |  |  |  |  |  |  |  |  |  |
| ABCC1, 2, 3, 4, 5, 6, 10, 11, 12, 13 subgroups |  |  |  |  |  |  |  |  |  |  |
| Cluster-41912.0_evgidbaidbtk80Loc41604 | K05665 | ABCC1 | ATP-binding cassette, subfamily C (CFTR/MRP), member 1 | | | | | | | |
| ABCG (White) subfamily |  |  |  |  |  |  |  |  |  |  |
| ABCG2, 3 subgroups |  |  |  |  |  |  |  |  |  |  |
| Cluster-29245.0_evgidbaidbtk80Loc80951 | K21396 |  | WHT | ATP-binding cassette, subfamily G (WHITE), eye pigment precursor transporter | | | | | | |
| Cluster-46327.0_evgidbad297756idbtk80Loc110721 | K21396 | WHT | ATP-binding cassette, subfamily G (WHITE), eye pigment precursor transporter | | | | | | | |
| ABC Transporters, Prokaryotic Type |  |  |  |  |  |  |  |  |  |  |
| ABC-2 type and other transporters |  |  |  |  |  |  |  |  |  |  |
| Heme transporter [MD:M00259] [OT] |  |  |  |  |  |  |  |  |  |  |
| Cluster-61241.0_evgidbad918078idbtk80Loc49165 | K02193 | ccmA | heme exporter protein A [EC:3.6.3.41] | | | |  |  |  |  |
| **Solute Carrier Family (SLC)** |  |  |  |  |  |  |  |  |  |  |
| SLC1: High-affinity glutamate and neutral amino acid transporter |  |  |  |  |  |  |  |  |  |  |
| Cluster-41979.0_evgidbad1384107idbtk80Loc40454 | K05614 | SLC1A3, EAAT1 | solute carrier family 1 (glial high affinity glutamate transporter), member 3 | | | | | | | |
| SLC3: Heavy subunits of the heteromeric amino acid transporters |  |  |  |  |  |  |  |  |  |  |
| Cluster-34386.0_evgidbaidbtk80Loc135797 | K14210 | SLC3A1, RBAT | solute carrier family 3 (neutral and basic amino acid transporter), member 1 | | | | | | | |
| SLC5: Sodium glucose cotransporter |  |  |  |  |  |  |  |  |  |  |
| Cluster-37026.0_evgidbad1396388idbtk80Loc53183 | K14388 | SLC5A8_12, SMCT | solute carrier family 5 (sodium-coupled monocarboxylate transporter), member 8/12 | | | | | | | |
| SLC6: Sodium- and chloride-dependent neurotransmitter transporter |  |  |  |  |  |  |  |  |  |  |
| Cluster-68794.0_evgidbaidbtk80Loc4688 | K05038 | SLC6A5S | solute carrier family 6 (neurotransmitter transporter, amino acid) member 5/7/9/14 | | | | | | | |
| Cluster-53752.0_evgidbad260152idbtk80Loc73001 | K05038 | SLC6A5S | solute carrier family 6 (neurotransmitter transporter, amino acid) member 5/7/9/14 | | | | | | | |
| SLC10: Sodium bile salt cotransporter |  |  |  |  |  |  |  |  |  |  |
| Cluster-33397.0_evgidbad909624idbtk80Loc40240 | K14343 | SLC10A3_5 | solute carrier family 10 (sodium/bile acid cotransporter), member 3/5 | | | | | | |  |
| SLC13: Human Na+-sulfate/carboxylate cotransporter |  |  |  |  |  |  |  |  |  |  |
| Cluster-49181.0_evgidbad61530idbtk80Loc65100 | K14445 | SLC13A2_3_5 | solute carrier family 13 (sodium-dependent dicarboxylate transporter), member 2/3/5 | | | | | | | |
| SLC16: Monocarboxylate transporter |  |  |  |  |  |  |  |  |  |  |
| Cluster-64912.0_evgidbaidbtk80Loc74119 | K08181 | SLC16A4 | MFS transporter, MCP family, solute carrier family 16 (monocarboxylic acid transporters), member 4 | | | | | | | |
| Cluster-31165.0_evgidbad505364idbtk80Loc76618 | K08189 | SLC16A13 | MFS transporter, MCP family, solute carrier family 16 (monocarboxylic acid transporters), member 13 | | | | | | | |
| SLC20: Type III Na+-phosphate cotransporter |  |  |  |  |  |  |  |  |  |  |
| Cluster-58611.0_evgidbad888700idbtk80Loc17549 | K14640 | SLC20A, PIT | solute carrier family 20 (sodium-dependent phosphate transporter) | | | | | | |  |
| SLC21/ASLCO: Organic anion transporter |  |  |  |  |  |  |  |  |  |  |
| Cluster-30061.0_evgidbad1375554idbtk80Loc31567 | K14352 | SLCO2B | solute carrier organic anion transporter family, member 2B | | | | | |  |  |
| Cluster-34595.0_evgidbad48191idbtk80Loc51650 | K14353 | SLCO3A | solute carrier organic anion transporter family, member 3A | | | | | |  |  |
| SLC22: Organic cation/anion/zwitterion transporter |  |  |  |  |  |  |  |  |  |  |
| Cluster-42547.0_evgidbaidbtk80Loc86803 | K08202 | SLC22A4_5, OCTN | MFS transporter, OCT family, solute carrier family 22 (organic cation transporter), member 4/5 | | | | | | | |
| SLC23: Na+-dependent ascorbic acid transporter |  |  |  |  |  |  |  |  |  |  |
| Cluster-58859.0_evgidbaidbtk80Loc16469 | K14611 | SLC23A1_2, SVCT1_2 | solute carrier family 23 (nucleobase transporter), member 1/2 | | | | | |  |  |
| SLC26: Multifunctional anion exchanger |  |  |  |  |  |  |  |  |  |  |
| Cluster-38869.0_evgidbad286087idbtk80Loc99009 | K14703 | SLC26A5, PRES | solute carrier family 26, member 5 | | | |  |  |  |  |
| SLC39: Metal ion transporter |  |  |  |  |  |  |  |  |  |  |
| Cluster-42848.0_evgidbaidbtk80Loc63308 | K14720 | SLC39A14, ZIP14 | solute carrier family 39 (zinc transporter), member 14 | | | | | |  |  |
| Cluster-38003.0_evgidbad901989idbtk80Loc32221 | K14720 | SLC39A14, ZIP14 | solute carrier family 39 (zinc transporter), member 14 | | | | | |  |  |
| Cluster-27244.0_evgidbad910044idbtk80Loc40682 | K14720 | SLC39A14, ZIP14 | solute carrier family 39 (zinc transporter), member 14 | | | | | |  |  |
| SLC44: Choline-like transporter |  |  |  |  |  |  |  |  |  |  |
| Cluster-53297.0_evgidbad446748idbtk80Loc13043 | K15377 | SLC44A2_4_5 | solute carrier family 44 (choline transporter-like protein), member 2/4/5 | | | | | | |  |
| SLC46: Folate transporter |  |  |  |  |  |  |  |  |  |  |
| Cluster-68726.1527_evgidbad467005idbtk80Loc36524 | K20840 | SLC46A3 | MFS transporter, PCFT/HCP family, solute carrier family 46, member 3 | | | | | | |  |
| Major Facilitator Superfamily (MFS) |  |  |  |  |  |  |  |  |  |  |
| **Organic acid transporters** |  |  |  |  |  |  |  |  |  |  |
| Monocarboxylate porter (MCP) family [TC:2.A.1.13] |  |  |  |  |  |  |  |  |  |  |
| Cluster-64912.0_evgidbaidbtk80Loc74119 | K08181 | SLC16A4 | MFS transporter, MCP family, solute carrier family 16 (monocarboxylic acid transporters), member 4 | | | | | | | |
| Cluster-31165.0_evgidbad505364idbtk80Loc76618 | K08189 | SLC16A13 | MFS transporter, MCP family, solute carrier family 16 (monocarboxylic acid transporters), member 13 | | | | | | | |
| Organic cation transporter (OCT) family [TC:2.A.1.19] |  |  |  |  |  |  |  |  |  |  |
| Cluster-42547.0_evgidbaidbtk80Loc86803 | K08202 | SLC22A4_5, OCTN | MFS transporter, OCT family, solute carrier family 22 (organic cation transporter), member 4/5 | | | | | | | |
| **Other Transporters** |  |  |  |  |  |  |  |  |  |  |
| Pores ion channels [TC:1] |  |  |  |  |  |  |  |  |  |  |
| Cluster-68725.0_evgidbad922445idbtk80Loc53811 | K19416 | yccA | modulator of FtsH protease | | |  |  |  |  |  |
| Cluster-51008.0_evgidbad493427idbtk80Loc63997 | K21894 | CYGB | cytoglobin | |  |  |  |  |  |  |
| Electrochemical potential-driven transporters [TC:2] |  |  |  |  |  |  |  |  |  |  |
| Cluster-27704.0_evgidbad34377idbtk80Loc37494 | K20989 | DUR3 | urea-proton symporter | | |  |  |  |  |  |
| Transmembrane electron carriers [TC:5] |  |  |  |  |  |  |  |  |  |  |
| Cluster-35310.0_evgidbad1366811idbtk80Loc22441 | K07306 | dmsA | anaerobic dimethyl sulfoxide reductase subunit A [EC:1.8.5.3] | | | | | |  |  |
|  |  |  |  |  |  |  |  |  |  |  |

**Urease metabolism genes in the genome of *Loripes orbiculatus* endosymbiont *candidatus* Thiodiazotropha endoloripes (Petersen et al. 2016)**

Petersen JM, Kemper A, Gruber-Vodicka H, Cardini U, van der Geest M, Kleiner M, Bulgheresi S, Mußmann M, Herbold C, Seah BKB *et al*: **Chemosynthetic symbionts of marine invertebrate animals are capable of nitrogen fixation**. *Nature microbiology* 2016, **2**:16195.

| **ID** | **Start** | **Stop** | **Size (nt)** | **Strand** | **Function** |
| --- | --- | --- | --- | --- | --- |
| fig\|6666666.392491.peg.57 | 59256 | 60557 | 1302 | + | Urea ABC transporter, substrate binding protein UrtA |
| fig\|6666666.392491.peg.58 | 60802 | 62421 | 1620 | + | Urea ABC transporter, permease protein UrtB |
| fig\|6666666.392491.peg.59 | 62425 | 63513 | 1089 | + | Urea ABC transporter, permease protein UrtC |
| fig\|6666666.392491.peg.60 | 63510 | 64352 | 843 | + | Urea ABC transporter, ATPase protein UrtD |
| fig\|6666666.392491.peg.61 | 64357 | 65052 | 696 | + | Urea ABC transporter, ATPase protein UrtE |
| fig\|6666666.392491.peg.62 | 65075 | 65254 | 180 | + | hypothetical protein |
| fig\|6666666.392491.peg.63 | 65323 | 66150 | 828 | + | Urease accessory protein UreD |
| fig\|6666666.392491.peg.64 | 66172 | 66810 | 639 | + | Urease gamma subunit (EC 3.5.1.5) |
| fig\|6666666.392491.peg.65 | 66814 | 68517 | 1704 | + | Urease alpha subunit (EC 3.5.1.5) |
| fig\|6666666.392491.peg.66 | 68551 | 69024 | 474 | + | Urease accessory protein UreE |
| fig\|6666666.392491.peg.67 | 69008 | 69694 | 687 | + | Urease accessory protein UreF |
| fig\|6666666.392491.peg.68 | 69707 | 70321 | 615 | + | Urease accessory protein UreG |

**Ionotropic glutamate receptors tree (10000 boot straps)**

Maximum likelihood tree constructed using IQ-TREE with peptide sequences in alignment published by Ramos-Vincente et al (2018). Peptide sequences were aligned using MAFT and model of evolution was select through the in-built test implemented in IQ-TREE. SH-aLRT /ultrafast bootstrap support values are indicated on the branches.

Ramos-Vicente D, Ji J, Gratacòs-Batlle E, Gou G, Reig-Viader R, Luís J, Burguera D, Navas-Perez E, García-Fernández J, Fuentes-Prior P et al: Metazoan evolution of glutamate receptors reveals unreported phylogenetic groups and divergent lineage-specific events. eLife 2018, 7:e35774.


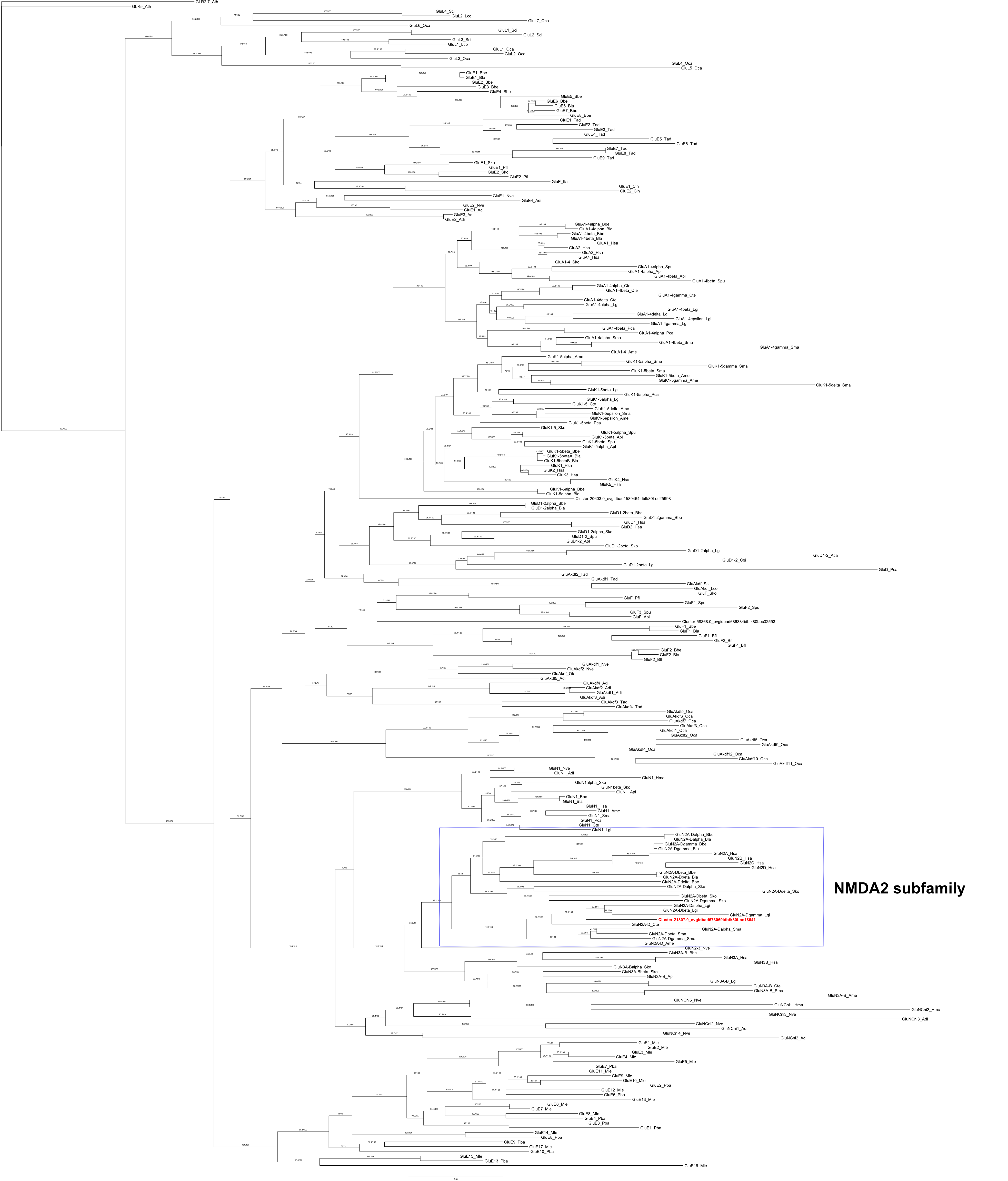

Supplement: Supplementary file 5 — Additional file 5. Trees and tables – Phylogenetic trees, table of the transporter genes annotated in the L. orbiculatus transcriptome, and table of the urease metabolism genes in the genome of Loripes orbiculatus endosymbiont candidatus Thiodiazotropha endoloripes [file 12864_2019_6177_MOESM5_ESM.docx]
